# Automatic Segmentation of Clinical Target Volumes for Post-Modified Radical Mastectomy Radiotherapy Using Convolutional Neural Networks

**DOI:** 10.3389/fonc.2020.581347

**Published:** 2021-02-16

**Authors:** Zhikai Liu, Fangjie Liu, Wanqi Chen, Xia Liu, Xiaorong Hou, Jing Shen, Hui Guan, Hongnan Zhen, Shaobin Wang, Qi Chen, Yu Chen, Fuquan Zhang

**Affiliations:** ^1^ Department of Radiation Oncology, Peking Union Medical College Hospital (CAMS), Beijing, China; ^2^ Department of Radiation Oncology, Sun Yat-sen University Cancer Center, Guangzhou, China; ^3^ MedMind Technology Co., Ltd., Beijing, China

**Keywords:** convolutional neural network, automatic segmentation, clinical target volume, breast cancer radiotherapy, clinical evaluation

## Abstract

**Background:**

This study aims to construct and validate a model based on convolutional neural networks (CNNs), which can fulfil the automatic segmentation of clinical target volumes (CTVs) of breast cancer for radiotherapy.

**Methods:**

In this work, computed tomography (CT) scans of 110 patients who underwent modified radical mastectomies were collected. The CTV contours were confirmed by two experienced oncologists. A novel CNN was constructed to automatically delineate the CTV. Quantitative evaluation metrics were calculated, and a clinical evaluation was conducted to evaluate the performance of our model.

**Results:**

The mean Dice similarity coefficient (DSC) of the proposed model was 0.90, and the 95th percentile Hausdorff distance (95HD) was 5.65 mm. The evaluation results of the two clinicians showed that 99.3% of the chest wall CTV slices could be accepted by clinician A, and this number was 98.9% for clinician B. In addition, 9/10 of patients had all slices accepted by clinician A, while 7/10 could be accepted by clinician B. The score differences between the AI (artificial intelligence) group and the GT (ground truth) group showed no statistically significant difference for either clinician. However, the score differences in the AI group were significantly different between the two clinicians. The Kappa consistency index was 0.259. It took 3.45 s to delineate the chest wall CTV using the model.

**Conclusion:**

Our model could automatically generate the CTVs for breast cancer. AI-generated structures of the proposed model showed a trend that was comparable, or was even better, than those of human-generated structures. Additional multicentre evaluations should be performed for adequate validation before the model can be completely applied in clinical practice.

## Introduction

Breast cancer is one of the most common malignant tumours in women. It was estimated that there were 2.1 million newly diagnosed female breast cancer cases, and 0.6 million cancer deaths in 2018 (1). Because radiotherapy and imaging quality technologies have advanced over the past decades, radiotherapy has become an effective treatment for breast cancer. A meta-analysis has shown that radiotherapy for postmastectomy patients can reduce locoregional recurrence, overall recurrence, and mortality (2). The precise delineation of the clinical target volume (CTV) is an essential step for accurate, individualized treatment. However, this task is time consuming and largely relies on the experience of oncologists. It is full of intra- and interobserver variability (3), which may obviously influence the efficacy of radiotherapy and the occurrence of complications (4). In addition, with the development of adaptive radiotherapy in recent years, clinicians are required to delineate the CTV accurately in a short time. Facing these new challenges, the application of artificial intelligence (AI) in radiotherapy may provide a feasible solution.

AI has been widely used in radiotherapy, including simulations (5), image segmentations (6, 7), treatment planning (8, 9), and quality assurances (10). It can increase the standardisation of working processes, lessen oncologists’ efforts and improve homogeneity. The convolutional neural network (CNN) has become the mainstream method for medical semantic segmentation because it has better performance than traditional atlas-based methods. It has been successfully applied in contouring several cancers’ CTVs, such as nasopharyngeal carcinomas (11), oropharyngeal carcinomas (12, 13), and rectal cancer (14). Men (15) constructed a very deep dilated residual network that could contour the CTVs automatically for patients who underwent conservative breast surgery. However, there is little research about CNNs being used for contouring the CTVs of patients who underwent modified radical mastectomies.

The autodelineation of CTVs is more challenging than that of organs at risk due to its low contrast visibility, potentially undetectable tumour regions, and strong dependence on the knowledge of clinicians. Specifically, the difficulties in contouring CTVs for postmastectomy patients include unclear boundaries and variability in the sizes and shapes of breasts. Since the segmentation performance of atlas-based methods depends on the accuracy of the image registration and the selected atlas (15), the delineation results are not satisfactory. Deep learning-based methods have the potential to obtain more accurate results. The U-Net architecture (16), proposed for the biomedical imaging community, has made significant contributions to the computer vision field. The encoder-decoder paradigm has been proven to be an effective way to conduct multilevel feature fusions. However, the network is not deep enough to represent high-level features, such as the structures that are of significant importance for breast CTV recognition. We therefore employed deeper convolution layers with the U-Net architecture as the backbone. To increase the network depth and ease the training of the network parameters simultaneously, the building blocks of the U-Net architecture were replaced with residual blocks of convolutional layers (17). We trained the U-Net and our proposed method under the same settings, and compared the predicted delineation results with the performance of the U-Net as the baseline.

## Materials and Methods

### Data and Pre-Processing

The CT data of 110 postmastectomy female patients were collected from March 2019 to July 2019 at Peking Union Medical College Hospital, Beijing, China. This study was approved by the Institutional Review Board of Peking Union Medical College Hospital. All patients met the indications for radiotherapy after modified radical mastectomies. 9130 CT slices were collected from those patients. Among the 110 patients, 54 received left chest wall radiotherapy, and the remainder received right side radiotherapy. All patients were scanned by a Philips Brilliance Big Bore CT scanner. Each CT image had a matrix size of 512 × 512 with 1.1543 mm × 1.1543 mm pixel spacing, and the thickness of each layer was 5 mm. The private information of patients was kept confidential during the data collection and processing. The delineation region of the chest wall CTV was defined according to the RTOG guidelines (18), which was from the caudal border of the clavicle head to the loss of apparent contralateral breast in a CT scan; the medial boundary was the sternal-rib junction, the lateral boundary was the mid-axillary line excluding the latissimus dorsi muscle, the anterior boundary was the skin and the posterior boundary was the rib-pleural interface. All the data were approved by two radiation oncologists who had more than 10 years of experience in breast cancer radiotherapy.

The intensity of the input images was clamped to −1024 HU and 1024 HU. A zero-mean normalization was applied to so that the different features had the same scale and to speed up the convergence of the weight parameters. “Ground truth (GT)” stands for the manually generated reference segmentation. The data were randomly divided into 3 groups. Eighty-eight cases were included in the training set, 11 cases were included in the validation set, and 11 cases were included in the testing set. In addition, we randomly selected 10 cases that had been applied in the clinic for further clinical evaluation.

### Network Architectures

**Figure 1:**
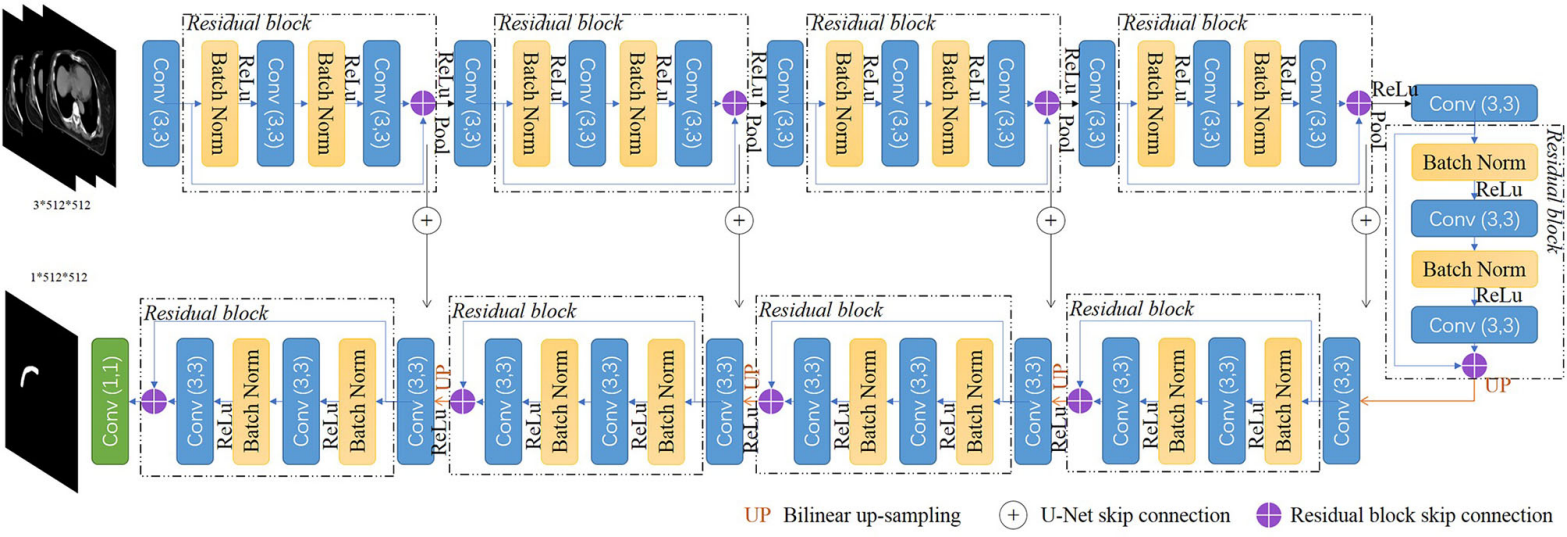
Overview of our proposed network.

We implemented a 2.5d fully CNN architecture to conduct the CTV mask segmentation task. The detailed network architecture is shown in **Figure 1**. A U-Net backbone architecture consisting of an encoding path and a decoding path was used. To obtain the 3D information of CT scans, and maintain contour continuity, the network was designed to assign three adjacent slices to three channels as the input. The building blocks were replaced with residual blocks to achieve consistent training as the network depth increased. Batch normalization (19), a linear transformation of the features, was used to reduce the covariance shift and accelerate the training procedure. The encoding path contained five convolutional layers and five residual blocks to gradually extract the features of the CTV region from low-level to high-level. In the decoder part, the upscaling was performed by using a nearest neighbors interpolation and was followed by a convolutional layer and a residual block. The encoding path and decoding path were combined together by a skip connection to concatenate the multilevel features and to take advantage of both the low-level and high-level information.

The original U-Net encodes relatively lower-level features such as edges and intensity contrasts. By replacing the convolutional layers with the residual blocks, our proposed model captures both low-level features and high-level features such as shapes, structures, and position relations to address the challenges of CTV recognition. As an efficient end-to-end training model, U-Net does not require a pretrained network and could be trained from scratch to achieve accurate segmentation results with very little labeled training data (16). Our proposed model uses the residual blocks to increase the network depth and eases the training of the network parameters simultaneously, and could also be trained from scratch with the amount of data we have.

A total of 99 patients’ CT scans were used for training and validation. All the CT slices were used. We did not use any pretrained models or transfer learning methods, and we trained our model from scratch. A weighted sum of cross-entropy loss and dice loss was used as the loss function. The output value of the model was in the range of 0 to 1. Pixels with output values larger than 0.5 were set as the foreground of the segmented mask. A contour extraction was applied to the foreground afterwards. The network was implemented using PyTorch 0.4.1 and Python 3.6 and trained on an NVIDIA GeForce GTX 1080 GPU with 8 GB memory. The entire network used the Adam optimizer with an initial learning rate of 0.0001, and was decayed by an exponential function with gamma 0.9 for every epoch. The total epoch number was 100.

### Quantitative Evaluation Metrics

The Dice similarity coefficient (DSC) (20) and Hausdorff distance (HD) (21) are commonly used for evaluating image segmentation performance. The DSC is defined as follows:

$$DSC\;(A,\;B)=\frac{2|A\cap B|}{\left|A|+|B\right|}$$

where A represents the predicted mask, and B is the GT mask. |A∩B| stands for the intersection of A and B.

The 95 HD is defined as:

95HD (A, B)=percentile [h(A,B)∪ h(B, A),95th]

$$h(A,\;B)=\underset{a\in A\;}{\text{max}\;}\underset{b\in B}{\text{min}}\;||a-b||$$

$$h(B,\;A)=\underset{b\in B\;}{\text{max}\;}\underset{a\in A}{\text{min}}\;||b-a||$$

||. || stands for the Euclidean norm of points A and B. A= {a_1_, a_2_, …, a_n1_} and B= {b_1_, b_2_, …, b_n2_} represent two finite point sets. 95HD indicates the 95^th^ percentile of mismatches between A and B (22). Both the DSC and 95HD were calculated at the two-dimensional level. Since our model was based on the U-Net model, we used the same data to train U-Net and then compared the DSC and 95HD with those of the proposed model.

Since the above evaluation does not completely reflect the segmentation quality, it is not clear whether it is significant for clinical practice. Therefore, it is also necessary for clinicians to evaluate the model.

### Clinical Evaluation

The evaluation was conducted by two other experienced clinicians, A and B, in our centre, who did not participate in the CTV contouring. Ten patients were selected randomly from the clinical work. The manual reference contours were separated into the GT group, while the corresponding contours generated by the proposed model belonged to the AI group. Then, the AI results and GT results of each case were randomly labeled 1 or 2. If AI was labeled 1, then GT was 2. Two clinicians were asked to score the 1 and 2 results, slice by slice, *via* a blind evaluation. **Table 1** shows the evaluation criteria. A score higher than 2 indicates that the contours were acceptable for clinical practice.

**Table 1:** The grading form used for CTV evaluation.

Score	Grade	Criteria
3	No revision	The segmentation is perfect and completely acceptable for treatment.
2	Minor revision	The segmentation needs a few minor edit but has no significant clinical impact without correction.
1	Major revision	The segmentation needs significant revision. Treatment planning should not proceed without contour correction.
0	Rejection	The segmentation is unacceptable and needs to be redrawn.

### Consistency Test

Ten slices from each CTV were randomly selected to mark the contours of both AI and GT simultaneously, and these slices constituted a dataset of 100 cases. Two clinicians blindly selected one contour that was better for clinical application. If the AI group was better, it was recorded as a positive result; otherwise, it was recorded as a negative result.

### Time Cost

The processing time was measured for AI, and pre- and post-AI assistance, in the delineation of CTV for postmodified radical mastectomy radiotherapy.

### Statistical Analysis

For the DSC and 95HD, a Wilcoxon signed-rank test was performed to verify whether the differences between our model and U-Net were statistically significant. The same test was performed to see if the differences in the scores given by the two clinicians were statistically significantly different. Furthermore, McNemar’s test and a consistency test were performed to check the evaluation consistency of the two clinicians.

## Results

### Segmentation Performance

**Figure 2:**
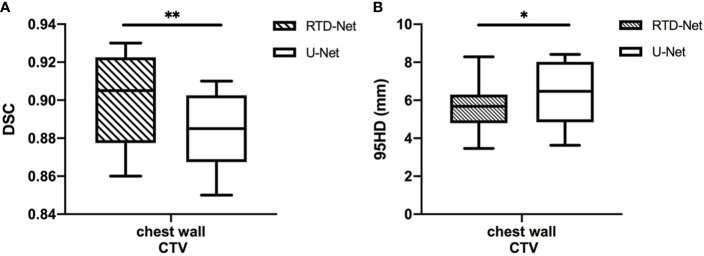
The box plot of the mean DSC **(A)** and the 95HD **(B)** results of proposed model and U-Net. *stands for P < 0.05, and **stands for P < 0.01.

The mean DSC of our proposed model is 0.90 ± 0.02, while that of U-Net is 0.88 ± 0.02 (*P*=0.007). The 95HD was 5.65 ± 1.29 mm in our model and 6.33 ± 1.63 mm in U-Net (*P*=0.037). The results of our model were not significantly different between the right side (DSC 0.90 ± 0.02, 95HD 5.94 ± 1.56) and the left side (DSC 0.90 ± 0.03, 95HD 5.31 ± 0.64, *P*=0.810 and 0.422, respectively). A detailed result is given in the **Supplementary Material Table 1**. The box plots are shown in **Figure 2**.

### Clinical Evaluation

**Figure 3:**
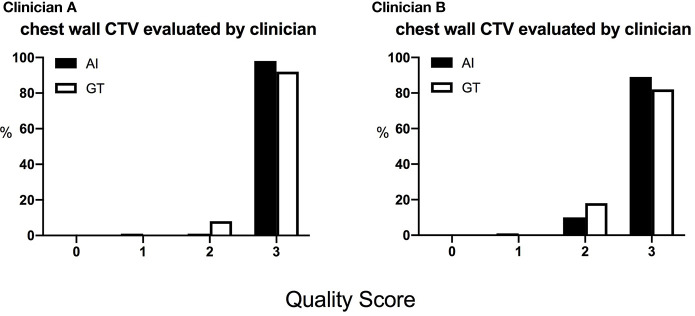
The distribution of the scores of the chest wall CTVs given by the two clinicians. The score is defined as 0, rejection; 1, major revision; 2, minor revision; 3, no revision.

**Figure 4:**
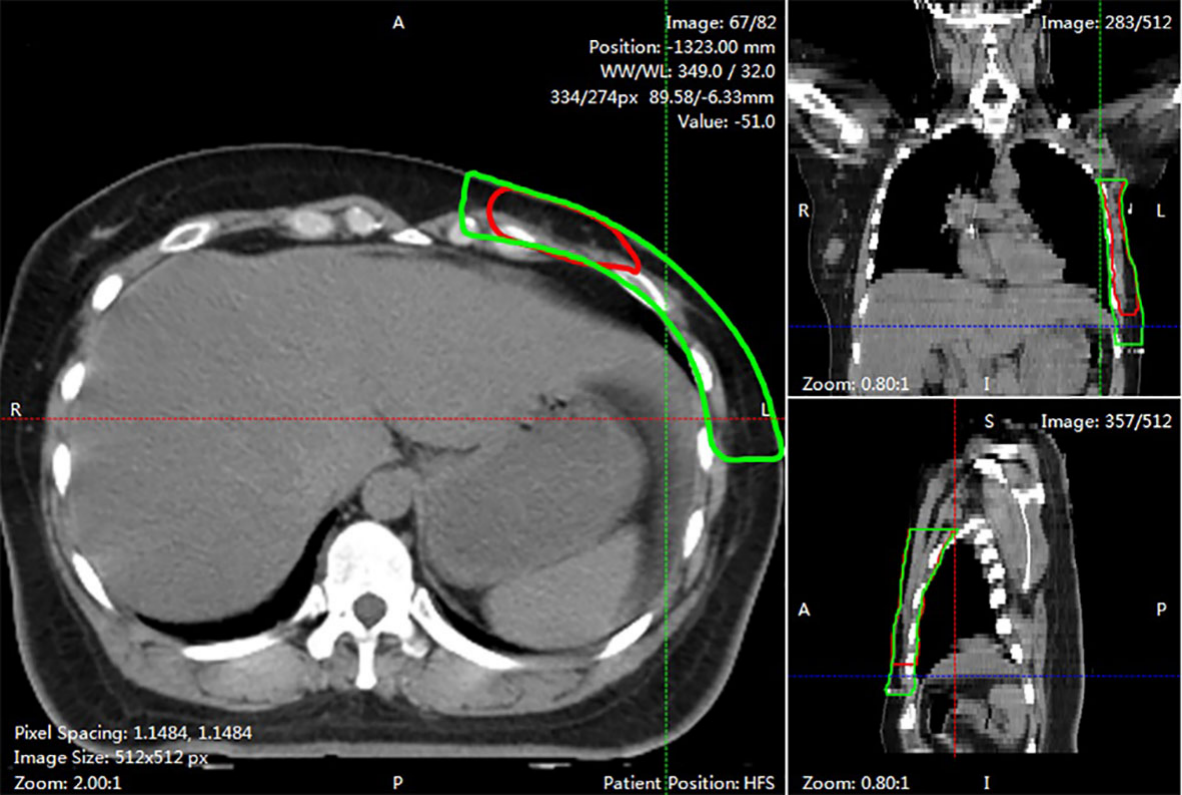
An example of segmented slice. This slide was graded 1 score by both two clinicians. The red line showed AI contours in three views. While the green line was GT contours.

**Figure 5:**
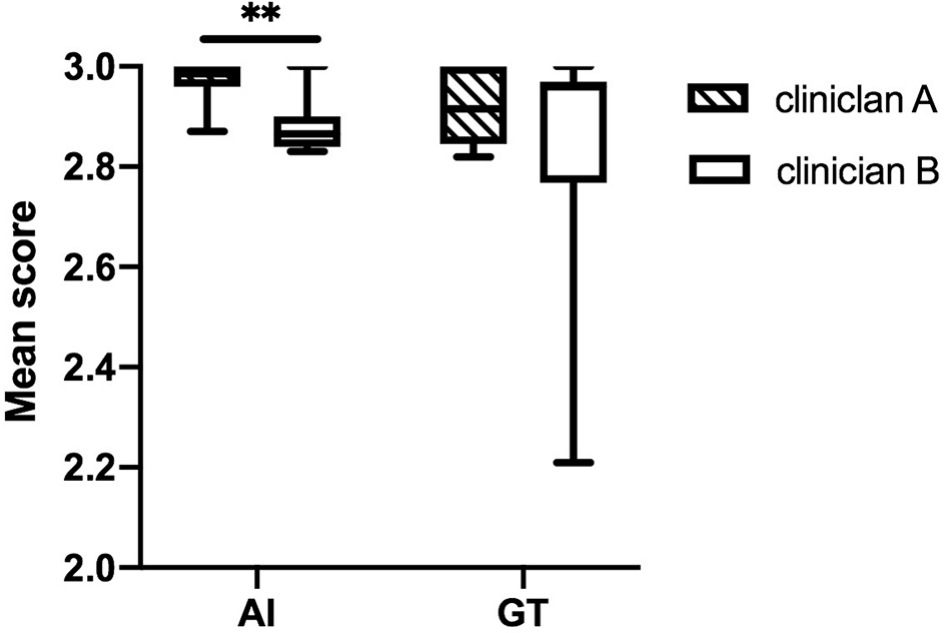
The box plots of the mean scores assigned by the two clinicians. The score differences are statistically significantly different between the two clinicians in the AI group. **stands for P < 0.01.

The DSC and 95HD values per patient are given in **Supplementary Material Table 2**. The evaluation results from the two clinicians are shown in **Table 2**, and the distribution of the clinical evaluation scores is shown in **Figures 2**, **3**. **Figure 4** shows an example segmented slice that is produced by the proposed model. If a score is higher than 2, this layer is acceptable for clinical applications. Therefore, the results given by clinician A show that 99.3% of the chest wall CTV slices from the AI group, and all the chest wall CTV slices from the GT group, can be accepted. The evaluation results from clinician B show that 98.9% of the chest wall CTV slices from the AI group, and all the chest wall CTV slices from the GT group, can be accepted. In addition, 9/10 of patients had all slices accepted by clinician A, while 7/10 could be accepted by clinician B. The score differences between the AI group and the GT group showed no statistically significant differences for either clinician (*P*=0.075 and *P*=0.444). The average scores given by clinician A were 2.97 (2.87–3.00) for the AI group, and 2.92 (2.82–3.00) for the GT group, while the average scores from clinician B were 2.88 (2.83–3.00) for the AI group, and 2.82 (2.21–3.00) for the GT group. The score differences were statistically significant between the two clinicians in the AI group (*P*=0.008) but there was no statistically significant difference in the GT group (*P*=0.721). The box plots of the mean scores are shown in **Figure 5**.

**Table 2:** The evaluation results from the two clinicians.

Score	Clinician A			Clinician B		
	AI group	GT group		AI group	GT group	
0	0 (0%)	0 (0%)		0 (0%)	0 (0%)	
1	2 (0.7%)	0 (0%)		3 (1.1%)	0 (0%)	
2	4 (1.4%)	23 (7.7%)		28 (9.8%)	54 (18.1%)	
3	278 (97.9%)	276 (92.3%)		253 (89.1%)	245 (81.9%)	
Total	284	299		284	299	
*P* value			0.075			0.444

AI, artificial intelligence; GT, ground truth.

### Consistency Evaluation

**Figure 6:**
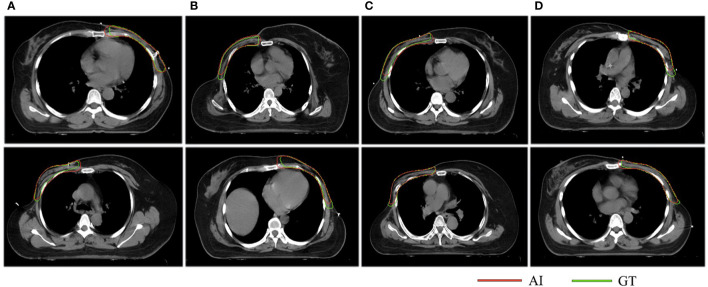
The results of the consistency test. The red lines represent the structures delineated by AI while the green lines stand for the structures contoured manually. Column **(A)** indicates that both clinicians think that AI is better, columns **(B, C)** suggest that the two clinicians have opposite opinions, and column **(D)** indicates that both clinicians think GT is better.

The evaluation results are shown in **Table 3**. Clinician A thinks that 60% of the CTV slices delineated by AI are better than the CTVs generated manually, while this number is 37% for clinician B. McNemar’s test is statistically significant (*P <*0.001), which means that the positive rates of the two clinicians are different. The Kappa consistency index was 0.259 (*P*<0.05), which means that the consistency between these two clinicians was poor. The evaluation results are shown in **Figure 6**.

**Table 3:** The results of the consistency evaluation.

Clinician B	Clinician A		Total
	Positive	Negative	
Positive	29	8	37
Negative	31	32	63
Total	60	40	100

### Timing Performance

The training time of the proposed model was 6 h using a GTX 1080 GPU. It takes more than 20 min for an oncologist to delineate a chest wall CTV completely. However, our model only needs 3.45 s to finish this task. With the assistance of AI, the contouring time was reduced to 10 min for an oncologist. This result indicates that this model can efficiently shorten the contouring time for clinicians.

## Discussion

To improve the working efficiency and reduce intra- and interobserver variability, we constructed a neural network model that can automatically delineate a chest wall CTV for breast cancer. We evaluated the segmentation performance and used a blind method to compare the delineation results with structures generated manually.

The delineation of the CTV is one of the most important steps in radiotherapy, and the accuracy is closely related to tumour control. Some studies have found that variations exist between different observers and different institutions, despite following the same contouring guidelines (3, 23). AI has been demonstrated to be an effective method to improve contouring accuracy and reduce variability (24). In regards to a postmastectomy CTV, the most important challenge is that some boundaries are not clear. The cranial and caudal planes of the contralateral breast are heterogeneous in different women, which will then affect CTV delineation. Since the lateral thoracic artery is destroyed after surgery, it is difficult to determine the position of the mid-axillary line without an anatomical reference mark. In addition, the RTOG guidelines recommend the interface between the ribs and pleura as the posterior boundary of the CTV. Most clinicians in our institution still use the RTOG guidelines, so the guidelines are also utilized in this study to ensure the proper implementation of the blind method.

Currently, there are very few studies in the field of chest wall CTV contouring with CNN models. The highest mean DSC was 0.84 when using atlas-based methods (25), while the mean DSC of our model was 0.90, with the potential for even better performance. However, the direct comparison of parameters is meaningless because the performance of the segmentation model largely depends on its ability to extract features, and in the consistency of the training data. Before moving into the next step of training, our data were strictly reviewed by experienced oncologists to minimize the variation in our data for further comparison.

From **Table 2**, we found that 97.9% of the CTV slices contoured by AI were accepted by clinician A and 89.1% by clinician B. Compared with human-generated structures, AI-generated structures are comparable or even better. Therefore, our model can be applied in clinical practice, and it may alleviate tedious workloads and reduce variations in the real world. According to **Figure 4**, we noticed that most slices that required minor or major corrections were located on cranial and caudal planes. The possible reason is that the delineation process needs to integrate information from multiple slices up and down, while the available information near cranial and caudal levels is limited, resulting in unsatisfactory contouring results. In addition, there was a statistically significant difference in scores between the two clinicians in the AI group, which means that AI delineations cannot meet all personal preferences.

In the consistency evaluation, AI-generated contours were directly compared with manual contours on the same slice. The results show that 29%, or even a higher proportion of AI-generated contouring were better, which suggests that the quality of CTV delineations could be improved with the assistance of AI. There are three possible reasons accounting for the poor consistency. First, the two clinicians had different understandings of the boundaries of the CTV, especially the medial and lateral boundaries. Second, clinicians may identify some implicit manual traces, and then choose the human-generated CTV as the better one. In addition, similar contours may lead to random selections.

There are three limitations in our study. First, the study was a single-centre study with a small sample size, which created a generalization problem. The results of our study can provide a reference for CTV delineation in patients with breast cancer. However, a multicentre evaluation with more cases should be performed in the future for better validation. Second, the model may not meet all clinicians’ preferences. Multiple institutions could achieve a consensus on delineation guidelines and provide a larger dataset, which will make the treatment in each centre more standardized. Finally, the grading process is subjective. Individual variations still need to be analysed in clinical practice.

## Conclusions

In this study, a novel CNN model is generated to delineate CTVs for postmastectomy patients automatically. The clinical evaluation results show that AI-generated structures trended towards being comparable, or even better, than human-generated structures. Our study provides a reference for CTV delineation in patients with breast cancer. We hope this work will help relieve clinicians from tedious contouring work, and minimize delineation variations from different centres. However, additional multicentre evaluations with more cases are needed before the model can be completely applied in clinical practice.

## Data Availability Statement

The raw data supporting the conclusions of this article will be made available by the authors, without undue reservation.

## Ethics Statement

The studies involving human participants were reviewed and approved by the institutional review board of Peking Union Medical College Hospital.

## Author Contributions

Study conception and design: ZL, FL, and FZ. Literature review: FL and WC. Data acquisition: FL, WC, XL, HZ, and HG. Model construction: QC, YC, and SW. Data approved: XH and FZ. Clinical evaluation: ZL and JS. Statistical analysis: FL, WC. Data interpretation: FL and XL. Manuscript preparation: ZL and FL. Manuscript review: ZL, FL, WC, XL, XH, JS, HZ, HG, and FZ. All authors contributed to the article and approved the submitted version.

## Funding

This work was supported by the Non-profit Central Research Institute Fund of Chinese Academy of Medical Sciences (grant 2019XK320014).

## Conflict of Interest

Author SW, QC and YC were employed by company MedMind Technology Co. Ltd.

The remaining authors declare that the research was conducted in the absence of any commercial or financial relationships that could be construed as a potential conflict of interest.
