# Supplementary material for: Automatic Segmentation of Clinical Target Volumes for Post-Modified Radical Mastectomy Radiotherapy Using Convolutional Neural Networks
Source: Front Oncol. 2021 Feb 16;10:581347. doi: 10.3389/fonc.2020.581347 (PMC7921705; doi:10.3389/fonc.2020.581347)
Supplement: Supplementary file 1 [file DataSheet_1.pdf]

Supplementary material

Supplementary table 1. Segmentation performance of test set.

No	Side	U-Net		Our proposed model	
		DSC	HD95	DSC	HD95
1	R	0.91	3.63	0.92	3.46
2	R	0.86	8.26	0.9	5.57
3	R	0.87	7.94	0.87	8.29
4	R	0.9	6.85	0.91	6.25
5	R	0.87	8.41	0.89	6.42
6	R	0.88	6.33	0.9	5.66
7	L	0.9	4.96	0.93	4.5
8	L	0.9	5.82	0.91	5.3
9	L	0.87	6.29	0.88	6.07
10	L	0.85	6.65	0.86	5.79
11	L	0.91	4.5	0.93	4.89
Average		0.88	6.33	0.90	5.65

Supplementary table 2. Clinical evaluation with our proposed model.

No.	Side	DSC	HD95
1	R	0.88	10.95
2	R	0.88	10.96
3	L	0.85	10.91
4	L	0.93	4.57
5	R	0.87	6.36
6	L	0.92	5.53
7	R	0.84	6.93
8	R	0.86	5.19
9	R	0.88	9.10
10	L	0.91	5.88
Average		0.88	7.64
